# The Influence of Hemocoagulation Disorders on the Development of Posttraumatic Cerebral Infarction and Outcome in Patients with Moderate or Severe Head Trauma

**DOI:** 10.1155/2013/685174

**Published:** 2013-08-04

**Authors:** Hao Chen, Li-Xia Xue, Yan Guo, Shi-Wen Chen, Gan Wang, He-Li Cao, Jiong Chen, Heng-Li Tian

**Affiliations:** ^1^Department of Neurosurgery, Shanghai 6th People Hospital, Shanghai Jiaotong University, 600 Yishan Road, Shanghai 200233, China; ^2^Department of Neurology, Shanghai 6th People Hospital, Shanghai Jiaotong University, Shanghai 200233, China

## Abstract

Posttraumatic cerebral infarction (PTCI) is a severe secondary insult of head injury and often leads to a poor prognosis. Hemocoagulation disorder is recognized to have important effects on hemorrhagic or ischemic damages. We sought to assess if posttraumatic hemocoagulation disorders were associated with cerebral infarction, and evaluate their influence on outcome among patients with moderate or severe head trauma. In this study, PTCI was observed in 28 (10.57%) of the 265 patients within the first week after injury. In multivariate analysis, the thrombocytopenia (odds ratio (OR) 2.210, 95% confidence interval (CI) 1.065–4.674), abnormal prothrombin time (PT) (OR 3.241, 95% CI 1.090–7.648), D-dimer (>2 mg/L) (OR 7.260, 95% CI 1.822–28.076), or disseminated intravascular coagulation (DIC) scores (≥5) (OR 4.717, 95% CI 1.778–12.517) were each independently associated with an increased risk of PTCI. Admission Glasgow Coma Scale (GCS) score, abnormal activated partial thromboplastin time (APTT) and fibrinogen, and D-dimer (>2 mg/L) and DIC scores (≥5) showed an independent predictive effect on poor outcome. In conclusion, recognition of this important treatable cause of PTCI and the associated risk factors may help identify the group at risk and tailor management of patients with TBI.

## 1. Introduction

Traumatic disorder of hemocoagulation is extremely common in traumatic brain injury (TBI) and is frequently associated with a poor outcome. The incidence is reported to vary between 15% and 100% [1]. It begins with the massive release of thrombin or tissue factor (TF), from damaged brain cells, following the activation of hemostatic mechanisms/coagulation pathway. The clinical manifestation of posttraumatic coagulopathy ranges from discrete abnormalities of hemostasis to very severe disorders, which provoke hemorrhagic and/or thrombotic damage of the central nervous system [2].

Posttraumatic cerebral infarction (PTCI) is a well-recognized complication in patients with head trauma. Recent reports and our previous study have demonstrated that intracranial hypertension, blunt cerebral vascular injuries, low Glasgow Coma Scores (GCS), hypotension, brain herniation, or decompression craniectomy may be risk factors for PTCI in patients with moderate or severe traumatic brain injury [3–5]. Although the association between consumptive coagulopathy and progressive hemorrhagic injuries and clinical outcome has been reported [6–11], the impact of hemocoagulative abnormalities on PTCI was seldom addressed. The present study was aimed at assessing if posttraumatic hemocoagulation disorders were associated with early PTCI and at delineating their influence on clinical outcome in patients with moderate or severe TBI. 

## 2. Clinical Materials and Methods

### 2.1. Patient Population

We conducted a retrospective study of all TBI patients admitted to the neurosurgery ward of the Sixth People's Hospital affiliated to Shanghai Jiaotong University between 2005 and 2010. For the present analysis, patients whose highest abbreviated injury score (AIS) was 3 or less (other than head injury) were considered to be isolated TBI cases. We included only isolated TBI patients admitted within 4 hours of injury with a GCS score of 12 or less. To avoid interfering factors, patients who suffered open injuries or had a history of cerebral infarction were excluded. Patients with known coagulation disorders, such as deep venous thrombosis or hemophilia, and those on anticoagulant therapies that could result in coagulopathies were also excluded. Those who deteriorated and died before a second computed tomography (CT) scan was performed or had ischemic lesions identified on first CT scan whose density remained unchanged during the neuroradiologic followup were not included in the study. In addition, we also excluded patients with systemic hypotension in the first 12 h after injury and/or treated with decompression craniectomy. Demographic data (gender and age), mechanism of injury, and admission GCS were documented when the patients arrived at the emergency room.

### 2.2. Patients Management

Axial CT scans of the head were obtained routinely on admission and thereafter. All patients had a CT scan repeated within 24 hours of admission. For some patients with diffuse brain injury, follow-up CT scans were scheduled on the 3rd day, 5th day, and 7th day after trauma. Intraventricular catheters were placed in some patients presenting with a GCS of 8 or below to permit continuous monitoring of intracranial pressure (ICP), as well as drainage of cerebrospinal fluid (CSF) when the ICP exceeded 20 mm Hg. Any clinical deterioration or increase in ICP indicated that another CT scan should be obtained. Magnetic resonance imaging (MRI) scanning was applied to further confirm the CT diagnosis or evaluate the questionable and not clearly delineated findings in CT images. Magnetic resonance angiography (MRA) scanning was applied to detect the infarct-related artery and the carotid or vertebral artery stenosis or occlusion. All the imaging studies were technically adequate and reviewed by an independent experienced neuroradiologist. Hypotension was defined as a systolic blood pressure lower than 90 mm Hg and/or diastolic pressure less than 40 mm Hg.

Diagnosis of PTCI was made on unenhanced CT scans according to the following criteria: (1) distinctly hypodense lesions within a defined cerebral vascular territory, involving the entire territory (complete) or part of it (incomplete); (2) hypodense lesions located in boundary zones between the defined cerebral vascular territories or situated in the terminal zones of perforating arteries within the deep white matter; (3) single or multiple hypodense lesions, unilateral, bilateral, or multifocal with marked borders without a precise localization in a vascular territory [12]. The diagnosis was made on MRI by visualization of a well-demarcated region, in an arterial vascular distribution, of low signal on T1-weighted images, high signal on conventional T2-weighted or fluid attenuation inversion recovery sequences, and of high signal on diffusion weighted images, indicating diminished perfusion, associated with a corresponding decrease in signal on the apparent diffusion coefficient map [1].

After admission, patients were taken either directly to the operating room for removal of intracranial hematomas or to the neurosurgery intensive care unit (NICU). Patients were evaluated and treated following the currently accepted international guidelines [1]. All patients were offered clinical and radiological evaluation at 3 months after injury via outpatient interviews and CT images of the brain. Outcome was evaluated using the Glasgow Outcome Scale (GOS) 3 months after the trauma: GOS 1 = death; GOS 2 = vegetative state; GOS 3 = severe neurological deficit; GOS 4 = mild neurological deficit; and GOS 5 = premorbid level of functioning or complete recovery [13]. Poor outcome was defined as a GOS score of ≤3.

### 2.3. Hemocoagulation Parameters Detection

A blood sample was taken from the antecubital vein for laboratory analyses immediately upon hospital admission. Platelet counts (normal values: 100–300 × 10^9^/L) and coagulation tests, including prothrombin time (PT; normal values: 11–14 s), activated partial thromboplastin time (APTT; normal values: 28–40 s), fibrinogen level (normal values: 2.0–4.0 g/L), and the concentration of D-dimer (normal values: 0–0.3 mg/L), were determined within the first 12 h of admission for all patients. All laboratory tests were performed in our clinical coagulation laboratory. A prolongation or shortness of 3 s and 10 s to the reference value of PT and APTT, respectively, was regarded as abnormality. 

Disseminated intravascular coagulation (DIC) score was calculated according to the scoring system of the International Society on Thrombosis and Haemostasis (ISTH) (Table 1) [14, 15]. Calculation of the score has been validated using either D-dimer or fibrin degradation products (FDP) titer, assigning points based on the degree of increase: none, moderate, or severe coagulation disturbances [15, 16]. Since FDP titer is not available in our laboratory, we had to modify the score by using D-dimer. Furthermore, the ISTH has labeled DIC scores ≥5 as “overt DIC”; therefore, each patient's DIC score was also analyzed as ≥5 and <5. 

### 2.4. Statistical Analysis

The statistical package Statistical Program for Social Sciences (version 17.0; SPSS, Inc., Chicago, IL 60606-6412, USA) was used for analyses. Association between PTCI and demographic factors, admission GCS, and hemocoagulative factors, at the univariate level, was evaluated using *χ* ^2^ test or, where appropriate, Fisher's exact test. Box and whisker plot was used to describe the general trends and association between GOS and clinical variables. Multivariate logistic regression model was used to evaluate the independent effect associated with PTCI and poor outcome at 3 months after head trauma. *P* values less than 0.05 were considered statistically significant. 

## 3. Results 

### 3.1. Characteristics of Head Injury

**Figure 1:**
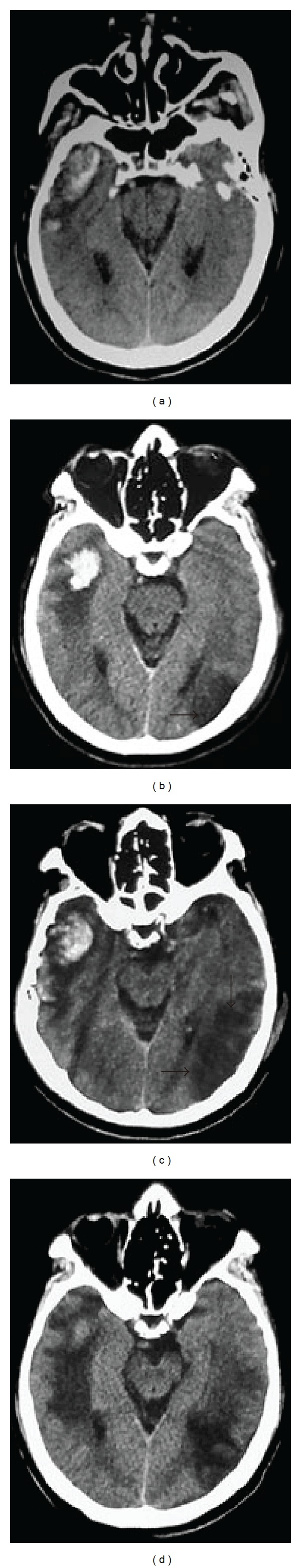
Axial CT images of a 35-year-old man with a GCS score of 8 at admission after a motor vehicle collision. Axial CT initially reveals right temporal lobe hematoma. The following CT performed 24 hours and 48 hours after trauma shows well-marginated low density in the left posterior cerebral artery (arrowhead), suggesting infarction (b)-(c). Repeat CT scan 2 weeks after trauma shows brain edema around the damaged areas (d).

From January 2005 to December 2010, 1057 patients with head trauma were admitted at our institution. Of these, 265 patients met inclusion criteria and were included in the study. The patients' ages ranged from 13 to 63 years (mean 32.4 years); 155 (58.5%) patients were male and 110 (41.5%) were female. The mechanisms of trauma included 97 motor vehicle collisions, 65 motorcycle accidents, 44 falls, 35 heavy strikes (patients who were hit by heavy objects such as bricks, sticks, or falling objects), and 24 cases of assaults. Of the 265 patients evaluated, 28 (10.57%) developed cerebral infarctions during the 1-week investigation period. Infarcts were observed most frequently in the area of the middle cerebral artery (9 cases), followed by the posterior cerebral artery (8 cases) and anterior cerebral artery (4 cases) areas. Infarcts in the pallidum, the hypothalamus, the thalamus, and vertebrobasilar area were seen in 2, 1, 1, and 2 cases, respectively. One patient displayed watershed cerebral infarction, with cortical and subcortical low densities crossing typical vascular territories. Carotid, vertebral artery stenosis, or occlusion was not found in these cases as shown above by MRA. An illustrative case shows that early PTCI of the occipital lobe results from compression of the posterior cerebral artery (PCA) (Figure 1). Three months after the injury, 48 (18.11%) patients had a GOS of 1, 8 (3.02%) had a GOS of 2, 33 (12.45%) a GOS of 3, 69 (26.04%) a GOS of 4, and 107 (40.38%) a GOS of 5. 

### 3.2. Univariate and Multivariate Analyses of Factors Related to PTCI

**Figure 2:**
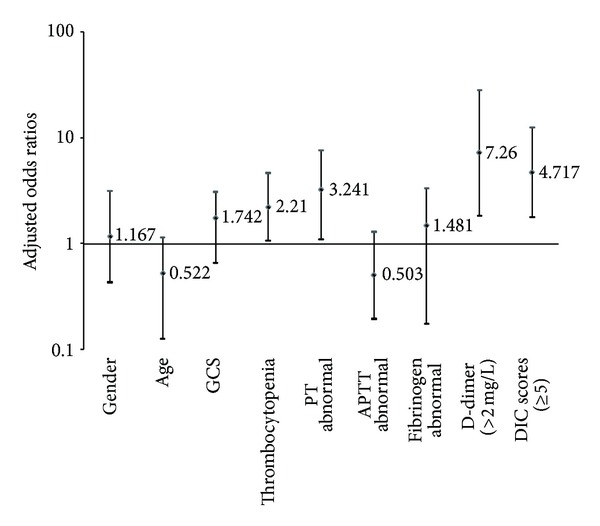
Adjusted odds ratios for early posttraumatic cerebral infarction risk factors in the multivariate models. PT: prothrombin time; APTT: activated partial thromboplastin time; DIC: disseminated intravascular coagulation.

Table 2 shows the demographic, clinical, and hemocoagulative characteristics of the patients and the incidence of early PTCI. The results of univariate analyses revealed that PTCI was associated at a significantly higher incidence with hematological abnormalities as follows: decreased platelet count (32.00%, *P* = 0.003), delayed PT (16.44%, *P* = 0.034) and APTT (18.03%, *P* = 0.043), low fibrinogen (21.74%, *P* = 0.001), and elevated D-dimer (14.61%, *P* = 0.001). Regarding the association between PTCI and the severity of TBI and coagulative disorders, a significant difference was observed between patients with GCS scores (3–5) (19.23%, *P* = 0.005) and DIC scores (≥5) (16.78%, *P* = 0.0002). The results of logistic regression studies are summarized in Table 3. Gender (OR 1.167, 95% CI 0.43–3.166), age (OR 0.522, 95% CI 0.126–1.153), admission GCS scores (OR 1.742, 95% CI 0.654–3.091), abnormal APTT (OR 0.503, 95% CI 0.194–1.3), and fibrinogen (OR 1.481, 95% CI 0.174–3.332) were not associated with PTCI, whereas the thrombocytopenia, abnormal PT, D-dimer (>2 mg/L), or DIC scores (≥5) were each independent predictors of early PTCI. Odds ratios are adjusted for all of the previous variables (Figure 2).

### 3.3. Univariate and Multivariate Analyses of Factors Predicting GOS

**Figure 3:**
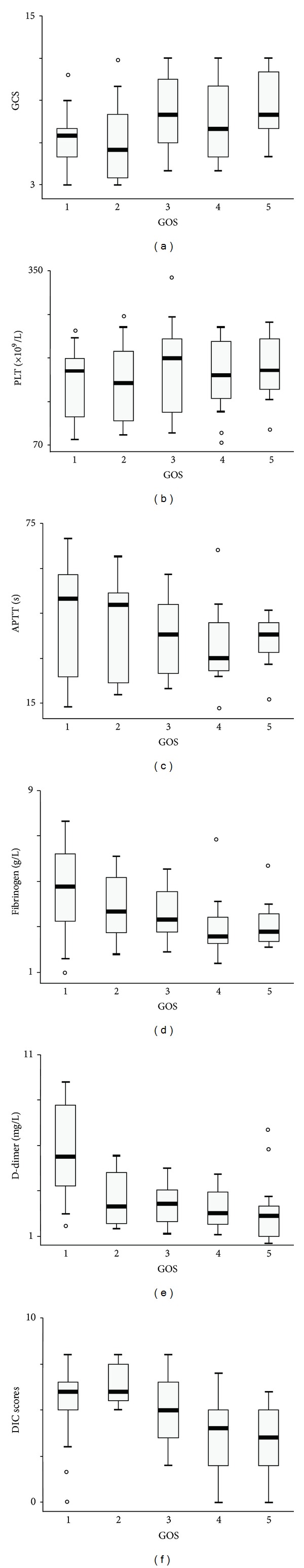
Box plots of GCS and of hemocoagulative factors at the time of hospital admission among patients with moderate or severe head injury. Data are shown by GOS 3 months after head trauma. GCS: Glasgow Coma Score; GOS: Glasgow Outcome Score; PLT: platelet; APTT: activated partial thromboplastin time; DIC: disseminated intravascular coagulation.

Table 4 presents GOS of the patients at 3 months after trauma. The results of univariate and multivariate analyses for determining the association between poor outcome and demographic factors, admission GCS, hemocoagulative factors, and PTCI are summarized in Table 5. At univariate regression analysis, the demographic factors and abnormal PT were not significantly associated with GOS and were, thus, excluded from the multivariate analysis. All of the other variables were significantly associated with unfavorable outcome at univariate analysis.  Figure 3 shows the box plots of GCS and the results of the coagulation tests in relation to the GOS. Patients who had longer APTT and higher D-dimer or fibrinogen level tend to have lower GOS scores, but higher initial GCS or PLT and lower DIC indicate an expected higher GOS. When simultaneously adjusting for all of these factors in the multivariate analysis, a significant association was found between poor outcome and initial GCS, abnormal APTT and fibrinogen, D-dimer (>2 mg/L), and DIC scores (≥5). 

## 4. Discussion 

Hemocoagulation disorder is a common and important consequence of TBI, with a reported incidence from 15 to 100% [1]. A number of literatures demonstrate that posttraumatic coagulopathy appears to be linked to secondary cerebral insults and often results in a poor clinical prognosis [8–11]. Cerebral infarction has been recognized as a potential secondary injury after brain trauma. It may occur secondary to focal mass effect, vascular impingement from herniated lobes, cerebral vasospasm, or venous congestion at craniectomy sites [17]. Furthermore, thromboembolism usually caused by posttraumatic coagulopathy is also a possible explanation for early PTCI. 

Previous studies have shown correlations between the development of this complication and intracranial hypertension, the presence of a blunt cerebral vascular injury, low systolic BP, brain herniation, and decompression craniectomy [3–5], but few studies have addressed hemocoagulative abnormalities and cerebral infarction following head trauma. Thus, we reviewed the records of 265 patients with moderate or severe head trauma to evaluate the relationship between them and their association with clinical outcome. 

In this study, 28 patients (10.57%) developed cerebral infarction within one week after injury, which is similar to the recent literature and our pervious study. Tawil et al. reported an overall 8% incidence of PTCI in a systematic review of 384 severe TBI patients [4]. The incidence was 11.90% in our previous cohort. Marino et al. reported that 19.1% had a confirmed PTCI in moderate and severe TBI, but only 89 patients were enrolled in their investigation [3]. 

The results of the multivariate analysis of our study show that patients with abnormalities of the platelet count, PT, D-dimer, and those with higher DIC scores were mostly at risk of developing cerebral infarction. Although there were strongly significant associations between early PTCI and initial GCS and abnormal APTT and fibrinogen at the univariate analysis, no association was observed in the final logistic regression model.

Previous studies of cerebral infarction in subjects without head trauma showed that increasing age was associated with cerebral infarction [18, 19]. However, we found no relation between age and early PTCI in our series. Additionally, the present study showed that male patients with moderate or severe TBI were more likely than females to develop cerebral infarction, but a statistically significant association was not found between gender and PTCI. Moreover, one point of separation from our previous study is that the admission GCS was not independently predictive of early PTCI in this present cohort. Differentiation of the study population and analyzed variables are the possible causes.

Association between coagulopathy and an increased incidence of ischemic stroke has been established in many prospective studies [20]. We also found that coagulation derangement was strongly associated with early PTCI. It has been hypothesized that the thrombotic-fibrinolytic imbalance after moderate or severe TBI may provoke an ischemic diathesis by the activation of hemostatic mechanisms and the inhibition of natural anticoagulation cascades [1, 2, 21, 22]. After head trauma, the release and upregulation of thromboplastin from the damaged parenchymal brain cells sequentially activate the procoagulant proteins of the extrinsic clotting pathway, which is represented by PT values. In addition, endothelial injury reinforces the coagulation cascade by launching intrinsic clotting factor and platelet aggregation. A prothrombogenic or hypercoagulation state, as reflected by D-dimer levels, leads to widespread intravascular deposition of fibrin, depletion of platelets and coagulation factors, and the development of intravascular microthrombosis [21]. Furthermore, fibrinolysic shutdown and posttraumatic inflammation accentuate the ischemia from thrombosis of small and midsize vessels and hence lead to cerebral infarction ultimately [22]. Among the markers of hemocoagulation disorders being studied, we found that thrombocytopenia, shortened or prolonged PT, and D-dimer (>2 mg/L) were independent predictors of early PTCI. Interestingly, it is generally recognized that the PT values should be shortened in the thrombotic diseases. However, massive consumption of the plasma coagulation factors during the course of thrombosis resulted in reducing the blood's ability to clot. It might be one of the main reasons for prolonged PT in the PTCI patients. It is worth noting that thrombocytopenia and D-dimer were also confirmed to be predictors of progressive hemorrhagic injury (PHI) after TBI in most studies. Nekludov et al. considered that the platelet dysfunction after severe head trauma most likely contributes to bleeding complications [23]. Allard et al. also found that platelet count was significantly correlated with intracranial hemorrhage progression in randomized controlled trial including 72 adult patients with blunt severe TBI [6]. Our previous study indicated that a D-dimer level of 5.00 mg/L was considered the cutoff point to prognose the possibility of PHI, with a sensitivity of 72.8% and a specificity of 78.8% [7]. We speculate that a strongly high level of D-dimer may indicate hyperfibrinolysis and a compensatory reaction to hypercoagulation that increase intracranial hemorrhagic risk. Thus, the effects of D-dimer on the development of cerebral infarction need to be further defined. 

Another important independent risk factor for suffering early PTCI is DIC. DIC is a frequent and very severe coagulation disorder following massive traumatic injuries, especially those involving the brain. Early development of DIC is induced by the burst of systemic thrombin generation, coupled with platelet hyperaggregability, hypothermia, and tissue hypoperfusion, resulting from the multifaceted derangement of hemostasis and impaired endogenous fibrinolysis [24]. This may damage microvasculature and ultimately contributes to thrombotic insults. Lozance et al. reported that 17 to 76% of patients with head injury developed DIC [25], and Chiaretti et al. reported that approximately 22.2% of children with severe head injury developed DIC, all of whom died and had shown severe brain edema [26]. In our study, 21.13% of the patients with moderate or severe head injury had developed DIC, and 26.79% of those with DIC developed early PTCI. 

Moreover, we also evaluated the association between clinical outcome and hemocoagulative abnormalities, initial GCS scores, and PTCI. The results of the multivariate analysis demonstrated that lower GCS, abnormal APTT and fibrinogen, elevated D-dimer, and higher DIC scores independently correlated to an increased risk of poor prognosis in patients with moderate to severe TBI, which is consistent with significant evidence in the literatures [8–11]. Selladurai et al. showed DIC scores to correlate inversely with GCS and on logistic regression analysis showed APTT, fibrin degradation products, and DIC scores to be independent predictors of outcome when controlling for GCS [8]. Kuo et al. demonstrated that coagulation state in head injury patients within 24 h after injury is of value in determining the outcome, and modified coagulopathy score > or = 4 is a good predictor to evaluate mortality rate [9]. In a recent study of 80 patients of moderate-to-severe head injury, Saggar et al. found that increased PT, FDP, and D-dimer values correlated with higher mortality, and high DIC scores predicted mortality with a high degree of accuracy [10]. Lustenberger et al. also concluded that the early coagulopathy occurring within 12 h after isolated severe TBI is a marker of increased morbidity and poor outcomes [11]. Our results showed that the development of PTCI was significantly associated with poor prognosis, whereas the association was not statistically significant at the multivariate level. Similarly, a recent study demonstrated that the cerebral infarction was the only independent factor predicting long term outcome of patients with moderate or severe TBI [3]. Thus, early identification of PTCI and endovascular intervention, as well as judicious use of procoagulant agents, may mitigate this important secondary injury. Further aggressive management, including surgical decompression in patients with large infarctions and refractory elevated ICP, should be considered as soon as possible in patients with cerebral infarction following head trauma.

The main limitation of this study is its retrospective nature. This might have introduced a significant bias in patient selection and data collection. Another important limitation of our assessment of PTCI risk factors is that we did not control for the secondary insults of hypoxia or brain edema and administration of hemostatic drugs. When considering the dramatic changes in posttraumatic coagulation mechanisms, we only investigated PTCI that occurred during the early stage (1 week) of TBI, so our results cannot be seen to represent its true incidence. Additionally, more detailed outcome measures could not be effectively collected in our study because of the retrospective design and difficulties with followup in our patient population. Finally, not all the patients received intraventricular catheters, so data on ICP was incomplete and excluded. Therefore, a further prospective controlled study of numerous clinical elements is clearly needed. 

## 5. Conclusions

Posttraumatic cerebral infarction is a relatively common complication in patients with head trauma. Cerebral infarction that develops early during the clinical course may produce a devastating outcome, with relatively high mortality and morbidity. Initial recognition by clinicians is often difficult because of the diverse clinical manifestations, the delay in presentation, and the associated brain injuries that accompany infarction. Early diagnosis and successful management of traumatic cerebral infarction require a high degree of clinical suspicion. The present study demonstrates that thrombocytopenia, abnormal PT, D-dimer (>2 mg/L), and DIC scores (≥5) represent important predictors of early PTCI in patients with moderate or severe head trauma. Additionally, hemocoagulation disorder occurring within 12 h after injury might serve as a marker of increased mortality and morbidity in patients with moderate and severe TBI. Those with initial lower GCS, abnormal APTT and fibrinogen, elevated D-dimer, and higher DIC scores had a statistically significant greater risk of a poor outcome. 

## Figures and Tables

**Table 1:** Components of the DIC score.

	0	1	2	3	Max points possible
Blood platelet count (×10^9^/L)	>100	51–100	≤50		2
Fibrinogen (g/L)	≥1.0	<1.0			1
D-dimer concentration (mg/L)	≤2.0		2.1–8.0	>8.0	3
Prolonged prothrombin time (s)	<3	3–6	>6		2

Total score					8

All individual components are summed for a total of eight possible points. If FDP titer is used instead of D-dimer, then cutoff values for the DIC score are 0 (≤5 g/L), 2 (6–40 g/L), and 3 (>40 g/L).

**Table 2:** Demographic characteristics and hemocoagulative abnormalities related to development of cerebral infarction within 1 week in patients with moderate or severe head trauma.

Characteristics of patients	No. of patients	PTCI (%)	*P* value
Gender			
Male	155	18 (11.61)	0.550
Female	110	10 (9.09)	
Age (years)			
<20	60	7 (11.67)	0.892
20–40	150	15 (10.00)	
>40	55	6 (10.91)	
Admission GCS scores			
3–5	52	10 (19.23)	0.005^b^
6–8	121	15 (12.40)	
9–12	92	3 (3.26)	
Platelet count (×10^9^/L)			
<100	25	8 (32.00)	0.003^b^
100–300	210	19 (9.05)	
>300	30	1 (3.33)	
PT (s)			
≤8	30	5 (16.67)	0.034^a^
8–17	162	11 (6.79)	
≥17	73	12 (16.44)	
APTT (s)			
≤18	27	4 (14.81)	0.043^a^
18–50	177	13 (7.34)	
≥50	61	11 (18.03)	
Fibrinogen (g/L)			
<2	69	15 (21.74)	0.001^b^
2–4	161	9 (5.59)	
>4	35	4 (11.43)	
D-dimer (mg/L)			
≤2	87	2 (2.30)	0.001^b^
>2	178	26 (14.61)	
DIC scores			
<5	209	13 (6.22)	0.000^b^
≥5	56	15 (26.79)	

PTCI: posttraumatic cerebral infarction; GCS: Glasgow Coma Score; PT: prothrombin time; APTT: activated partial thromboplastin time; DIC: disseminated intravascular coagulation.

^a^ *P* < 0.05 marked statistical significance.

^b^ *P* < 0.01 more marked statistical significance.

**Table 3:** Multivariate logistic regression analysis of the association between demographic and hemocoagulative characteristics and early PTCI.

Clinical factors	OR value	95% CI	*P* value
Thrombocytopenia	2.210	1.065–4.674	0.024
PT abnormal	3.241	1.090–7.648	0.013
D-dimer (>2 mg/L)	7.260	1.822–28.076	0.016
DIC scores (≥5)	4.717	1.778–12.517	0.002

OR: odds ratio; CI: confidence interval; PT: prothrombin time; DIC: disseminated intravascular coagulation.

**Table 4:** Demographic characteristics, hemocoagulative abnormalities, GCS, PTCI, and outcome of 265 patients with moderate or severe head trauma.

Characteristics of patients	*n*	GOS 3 months after head trauma (%)				
		GOS 1	GOS 2	GOS 3	GOS 4	GOS 5
Gender						
Male	155	30 (19.35)	5 (3.26)	22 (14.19)	38 (24.52)	60 (38.71)
Female	110	18 (16.36)	3 (2.73)	11 (10.00)	31 (28.18)	47 (42.73)
Age (years)						
<20	60	5 (8.33)	2 (3.33)	9 (15.00)	14 (23.33)	30 (50.00)
20–40	150	31 (20.67)	5 (3.33)	18 (12.00)	37 (24.67)	59 (39.33)
>40	55	12 (21.82)	1 (1.82)	6 (10.91)	18 (32.73)	18 (32.73)
Admission GCS scores						
3–5	52	13 (25.00)	4 (7.69)	6 (11.54)	19 (36.54)	10 (19.23)
6–8	121	2 (26.45)	3 (2.48)	13 (10.74)	28 (23.14)	45 (37.19)
9–12	92	3 (3.26)	1 (1.09)	14 (15.22)	22 (23.91)	52 (56.52)
Platelet count (×10^9^/L)						
<100	25	4 (16.00)	2 (8.00)	5 (20.00)	6 (24.00)	8 (32.00)
100–300	210	44 (20.95)	6 (2.86)	24 (11.43)	54 (25.71)	82 (39.05)
>300	30	0 (0.00)	0 (0.00)	4 (13.33)	9 (30.00)	17 (56.67)
PT (s)						
≤8	30	1 (3.33)	2 (6.67)	3 (10.00)	13 (43.33)	11 (36.67)
8–17	162	33 (20.37)	2 (1.23)	16 (9.88)	42 (25.93)	69 (42.59)
≥17	73	14 (19.18)	4 (5.48)	14 (19.18)	14 (19.18)	27 (36.99)
APTT (s)						
≤18	27	3 (11.11)	2 (7.41)	3 (11.11)	9 (33.33)	10 (37.04)
18–50	177	38 (21.47)	3 (1.69)	25 (14.12)	39 (22.03)	72 (40.68)
≥50	61	7 (11.48)	3 (4.92)	5 (8.20)	21 (34.43)	25 (40.98)
Fibrinogen (g/L)						
<2	69	11 (15.94)	3 (4.35)	8 (11.59)	21 (30.43)	26 (37.68)
2–4	161	35 (21.74)	4 (2.48)	21 (13.04)	35 (21.74)	66 (40.99)
>4	35	2 (5.71)	1 (2.86)	4 (11.43)	13 (37.14)	15 (42.86)
D-dimer (mg/L)						
≤2	87	5 (5.75)	2 (2.30)	13 (14.94)	26 (29.89)	41 (47.13)
>2	178	43 (24.16)	6 (3.37)	20 (11.24)	43 (24.16)	66 (37.08)
DIC scores						
<5	209	26 (12.44)	5 (2.39)	18 (8.61)	58 (27.75)	102 (48.80)
≥5	56	22 (39.29)	3 (5.36)	15 (26.79)	11 (19.64)	5 (8.93)
Combined with PTCI						
Yes	28	10 (35.71)	3 (10.71)	5 (17.86)	6 (21.43)	4 (14.29)
No	237	38 (16.03)	5 (2.11)	28 (11.81)	63 (26.58)	103 (43.46)

GOS: Glasgow Outcome Score; GCS: Glasgow Coma Score; PT: prothrombin time; APTT: activated partial thromboplastin time; DIC: disseminated intravascular coagulation; PTCI: posttraumatic cerebral infarction.

**Table 5:** Association between demographic and hemocoagulative characteristics, GCS, PTCI, and poor outcome (GOS ≤ 3) among 265 patients with moderate or severe head trauma.

Clinical factors	Univariate analysis			Multivariate analysis		
	OR	95% CI	*P* value	OR	95% CI	*P* value
Gender (male)	1.418	0.838–2.397	0.193	NI	—	—
Age (20–40 ys)	0.833	0.565–1.288	0.525	NI	—	—
Admission GCS scores (3–5)	3.837	1.276–7.644	0.001^b^	5.953	1.351–13.013	0.009^b^
Thrombocytopenia	1.632	1.108–3.960	0.025^a^	2.276	0.473–3.438	0.630
PT abnormal	1.272	0.757–2.140	0.364	NI	—	—
APTT abnormal	1.426	1.218–3.833	0.013^a^	1.418	1.205–3.853	0.016^a^
Fibrinogen abnormal	2.372	1.193–4.718	0.003^b^	3.327	1.160–7.668	0.002^b^
D-dimer (>2 mg/L)	2.472	1.263–4.845	0.012^a^	2.489	1.242–4.989	0.047^a^
DIC scores (≥5)	3.403	1.221–7.734	0.003^b^	4.102	2.047–10.223	0.000^b^
Presence of PTCI	3.281	1.238–8.699	0.017^a^	2.542	0.931–6.941	0.069

OR: odds ratio; CI: confidence interval; NI: not included in the multivariate regression analysis; GCS: Glasgow Coma Score; PT: prothrombin time; APTT: activated partial thromboplastin time; DIC: disseminated intravascular coagulation; PTCI: posttraumatic cerebral infarction.

^a^ *P* < 0.05 marked statistical significance.

^b^ *P* < 0.01 more marked statistical significance.
